# Single-port laparoscopic excision of choledochal cysts in neonates versus older infants and children: a comparative safety and feasibility study from a single-center 5-year experience

**DOI:** 10.3389/fped.2026.1832330

**Published:** 2026-06-09

**Authors:** Bin Yan, Junkai Xiao, Pengjian Zou, Qiuming He, Xisi Guan, Xiaoli Xie, Junjie Wang, Wenfeng Tang, Jiakang Yu, Wei Zhong, Zhe Wang

**Affiliations:** 1 Guangzhou Women and Children’s Medical Center, Guangzhou Medical University, Guangzhou, China; 2 Guangzhou Medical University, Guangzhou, China

**Keywords:** choledochal cyst, minimally invasive surgery, neonatal surgery, Roux-en-Y hepaticojejunostomy, single-port laparoscopy

## Abstract

**Objective:**

This study compared the feasibility, safety, and efficacy of single-port laparoscopic choledochal cyst excision with Roux-en-Y hepaticojejunostomy (SPCH) between neonates and older infants and children to determine whether early surgical intervention in the neonatal period carries additional operative risks or offers comparable outcomes to the current standard of care.

**Methods:**

A retrospective comparative cohort study was conducted on children who underwent SPCH between March 2020 and June 2024. Perioperative data, including operative time, blood loss, complications, and postoperative hospitalization were analyzed.

**Results:**

A total of 70 patients were included, comprising 21 neonates (≤28 days) and 49 older infants/children (>28 days). Neonates had larger cyst diameters without statistical significance (5.49 vs. 4.49 cm, *P* = 0.052), higher rates of prenatal detection (85.7% [18/21] vs. 49.0% [24/49], *P* = 0.004), whereas older infants/children more frequently presented with pancreaticobiliary maljunction (44.9% [22/49] vs. 4.8% [1/21], *P* = 0.001).Operative outcomes were comparable, with no significant differences in operative time (230.33 vs. 256.43 min) or blood loss (6.43 vs. 6.86 mL). Neonates had no small diameter hepaticojejunostomy anastomoses (0.0% [0/21] vs. 32.7% [16/49], *P* = 0.003) but longer postoperative stays (8.24 vs. 6.69 days, *P* = 0.04). Complication rates were similar (4.8% [1/21] vs. 2.0% [1/49], *P* = 0.53).

**Conclusion:**

For neonatal CDC, SPCH is a practical and secure solution offering cosmetic benefits without increasing procedural risks. Neonatal anatomical features facilitate technical success, though heightened postoperative surveillance requirements account for longer hospitalization.

## 1 Introduction

Choledochal cyst (CDC) is one of the most common congenital biliary malformations in East Asia (1/10,000–1/15,000 births) (1). Laparoscopic cyst excision with Roux-en-Y hepaticojejunostomy has become the preferred surgical approach for CDC (2–6). SPCH performed through a single umbilical incision, provides additional cosmetic benefits and potentially reduced postoperative pain. However, SPCH faces technical challenges including restricted visual field and impaired instrument triangulation, which may prolong operative time and increase procedural risks in inexperienced hands.Applying SPCH to treat CDC in neonates is a challenge (7–11).

The optimal timing of surgical intervention for CDC in neonates remains among the most debated questions in pediatric hepatobiliary surgery (12, 13). Current guidelines recommend delaying elective repair until at least 3 months of age, primarily reflecting concerns about neonatal hepatic immaturity, biliary hypoplasia, increased anesthesia vulnerability, and the technical challenge of fashioning a precise hepaticojejunostomy in small-caliber ducts. Yet this conservative approach cannot be indiscriminately applied to neonates with large cysts (≥5 cm) or those presenting with symptoms, who face considerable risks of spontaneous perforation, severe cholangitis, and progressive hepatic fibrosis while awaiting operation. Observational data increasingly indicate that early surgical intervention may offer tangible therapeutic advantages in appropriately selected neonatal cases (14–16).

To date, evidence directly comparing neonatal vs. older infants and children SPCH outcomes remains scarce, and most published series focus exclusively on older infants or children. Consequently, it remains unknown whether the perceived technical disadvantages of neonatal anatomy translate into measurable differences in operative outcomes, or whether neonatal-specific factors (such as more elastic ductal tissue and larger relative cyst size) may, in fact, offer unappreciated technical advantages.

Therefore, we designed this comparative cohort study to benchmark neonatal SPCH outcomes against a concurrent older infants and children control group treated by the same surgical team using identical techniques. Our hypothesis was that in experienced hands, neonatal SPCH would achieve comparable operative safety and efficacy to the standard older infants and children population, thereby providing evidence to support early surgical intervention in selected high-risk neonates without the need for delayed repair.

## 2 Methods

### 2.1 Study population and data collection

This research was approved by the Ethics board of Guangzhou Women and Children's Medical Center [approval No. (2022) 083A01]. Written informed consent was provided. All the experimental protocols involving human data were conducted in accordance with national/international/institutional guidelines or the Declaration of Helsinki in the manuscript.

Patients meeting the following criteria were included: (1) diagnosis of CDC was confirmed by magnetic resonance cholangiopancreatography (MRCP); (2) clinical presentation met the CDC surgical indications (symptomatic patients under 3 months with abdominal pain, obstructive jaundice, cholangitis, or cyst diameter ≥5 cm; asymptomatic patients with cysts <5 cm underwent elective surgery after 3 months of age); (3) written informed consent for SPCH was obtained from parents. (4) SPCH was performed between March 1st, 2020 and June 1st, 2024. Exclusion criteria were: (1) missing preoperative or perioperative data, or loss to follow-up; (2) presence of other congenital anomalies requiring simultaneous surgical correction; and (3) conversion to multiport laparoscopy or open surgery.

In the postoperative follow-up protocol, patients were scheduled for clinical evaluations at one and six months and annually following surgical intervention. The initial postoperative assessment included evaluation of hepatobiliary function, quantification of total and direct bilirubin, pancreatic enzyme profiling (serum amylase and lipase), and hepatobiliary ultrasonography. A minimum follow-up duration of 14 months was maintained for all cases to permit rigorous evaluation of short-term surgical outcomes and postoperative complications. Patients were divided into two groups according to their operative age: The neonatal group (age ≤ 28 days) and the older infants and children group (age > 28 days). The patients' preoperative clinical data, such as age, gender, Todani's classification, pancreaticobiliary maljunction (PBM), symptoms, prenatal diagnosis details, and cyst diameters, were gathered. Perioperative information, including the surgical duration, amount of blood loss, postoperative LOS, drainage placement, and postoperative complications, was documented.

### 2.2 Surgical indication and technique

Immediate surgery is indicated for patients under 3 months of age with symptoms including abdominal pain, obstructive jaundice, cholangitis, or cysts' largest diameter ≥5 cm. Asymptomatic patients with cysts <5 cm undergo elective surgery after reaching 3 months of age.

**Figure 1:**
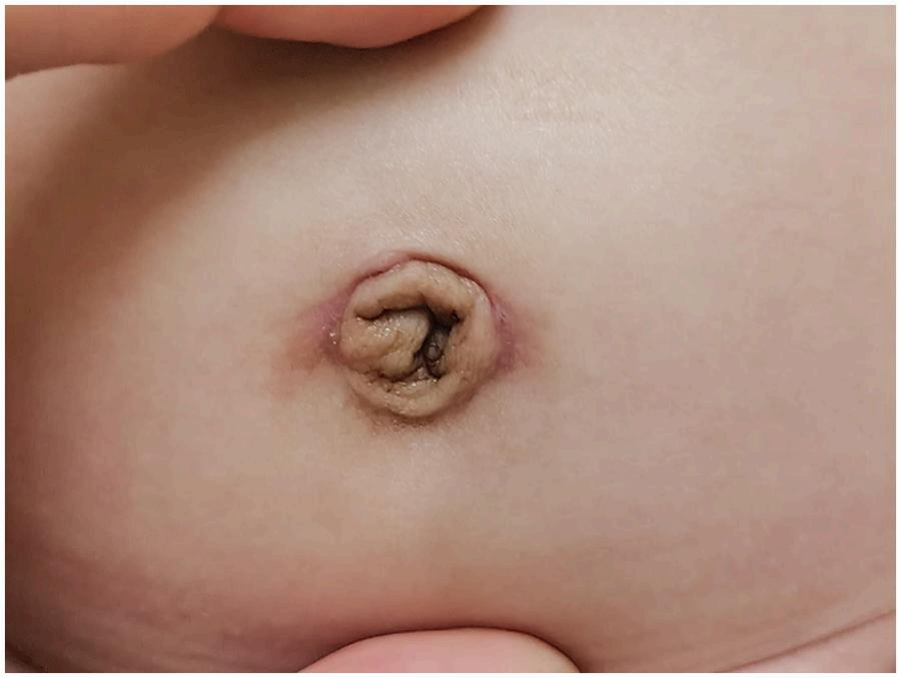
Scar of the 2–3 cm umbilical incision of SPCH.

Patients were placed in supine, an arc-shaped incision was made at the supraumbilical skin fold (Figure 1). A 35 mm oval single-port (Surgaid IIIA 3B-35 × 100, Xiamen Sanda Medical) was inserted. The pneumoperitoneum pressure was set to 8mmHg. Conventional 5 mm 30-degree laparoscope and 3 mm instruments were used. CDC dissection was performed according to the standard procedure; the pancreatic segment of the common bile duct was resected to its junction with the pancreatic duct. For neonates/infants with large CDC extending caudally to the umbilical level, distal CDC dissection was initially performed through the umbilical port under direct visualization, then the proximal cyst separation was finished under laparoscopy. Traction sutures are used to retract the gallbladder bed or CDC to the abdominal wall to improve exposure. Roux-en-Y reconstruction was completed through the single-port laparoscopy, no routine drainage tube was left after surgery unless in cases with risks of postoperative bleeding or bile leakage.

### 2.3 Outcome measures

The primary outcome measures were: (1) operative success rate (completion of SPCH without conversion); (2) operative time (minutes, from skin incision to closure); (3) estimated blood loss (mL, calculated by suction bottle volume plus gauze weighing method); and (4) postoperative complications within 30 days (Clavien-Dindo grade ≥ II, with specific attention to bile leakage, bleeding, and anastomotic stricture requiring reoperation). Secondary outcome measures included: (1) postoperative length of hospital stay (days); (2) drainage tube placement rate; (3) small-diameter hepaticojejunostomy anastomosis rate (defined as common hepatic duct diameter <5 mm requiring ductoplasty); and (4) long-term complications during follow-up (anastomotic stricture, cholangitis, adhesive bowel obstruction).

### 2.4 Statistical analysis

Statistical analyses were performed using GraphPad Prism 9 Version 9.0.0 (GraphPad Software, San Diego, CA, USA). For neonatal vs. older infants and children subgroup comparisons, non-normally distributed continuous variables (cyst diameter, operative time, blood loss, postoperative stay) were analyzed using the Mann–Whitney *U*-test, while categorical variables (symptom incidence, complication rates, drainage placement, small-diameter anastomosis) were assessed via Chi-square or Fisher's exact tests. Statistical significance was defined as *P* < 0.05. Missing data were addressed with pairwise deletion.

## 3 Results

### 3.1 Patient demographic characteristics and preoperative evaluation

Between March 15, 2020, and May 1, 2024, 70 patients underwent laparoscopic cyst excision and Roux-en-Y hepatic enterostomy by a single surgeon, there were 21 neonates and 49 infants and children. Preoperative characteristics between two groups showed no significant differences in genders, cyst type, or jaundice incidence. Significantly, the non-neonates had significantly more abdominal pain (0/21 vs. 20/49, *P* *=* 0.0005). Significantly less PBM was diagnosed in the neonatal group than in the older infants and children group (1/21 vs. 22/49, *P* *=* 0.001), There were significantly more prenatal diagnosed CDC in neonates than the non-neonates (18/21 vs. 24/49, *P* = 0.004). The average cyst maximum diameter was larger in neonates, but the difference was not statistically significant (5.49 cm vs. 4.49 cm, *P* *=* 0.052) (Table 1).

**Table 1:** Comparison of clinical characteristics between the neonatal group and the older infants/children group.

Characteristics	Neonatal group (*n* = 21)	Older infants/children group (*n* = 49)	*P*-value
Gender (male/female)	8/13	15/34	0.54
Todani classification			
Ia	10	15	0.17
Ib	4	7	0.62
Ic	3	11	0.43
Iva	4	14	0.40
Ivb	0	2	0.35
PBM	1	22	0.0011
Symptoms			
Abdominal pain	0	20	0.0005
Jaundice	9	16	0.41
Prenatal diagnosis	18	24	0.004
Diameter of cyst (cm)	5.49	4.49	0.052

PBM, Pancreaticobiliary maljunction.

### 3.2 Intraoperative outcomes

All patients successfully underwent SPCH. No significant differences were observed in operative time (230.33 min vs. 256.43 min), intraoperative blood loss (6.43 mL vs. 6.86 mL), or hepatic ductoplasty rate (2/21 vs. 9/49). The small diameter hepaticojejunostomy anastomosis (common hepatic duct diameter <5 mm before ductal plasity) was encountered significantly more often in older infants and children group (0/21 vs. 16/49, *P* = 0.003) (Table 2).

**Table 2:** Comparison of surgical data between the neonatal group and the older infants/children group.

Surgical outcomes	Neonatal group (*n* = 21)	Older infants/children group (*n* = 49)	*P*-value
Operative duration (min)	230.33	256.43	0.10
Blood lost (ml)	6.43	6.86	0.87
Hepatic ductoplasty	2	9	0.34
Small diameter anastomosis	0	16	0.003
Postoperative length of stay (days ± SD)	8.24 ± 2.65	6.69 ± 2.79	0.04
Draining tube placing	2	6	0.74
Post operative complications	1	1	0.53

### 3.3 Postoperative recovery and complications

Patients were followed up for 36.67 months (median 35 months, maximum 64 months), two patients experienced postoperative complications, one with hepaticojejunostomy stenosis and the other with common hepatic duct stenosis proximal to the hepaticojejunostomy. Both complications were resolved through reoperation. postoperative parameters showed no significant differences in drainage tube placement rate (2/21 vs. 9/49) or complication rates (1/21 vs. 1/49) in both groups. The neonatal group had significantly prolonged postoperative hospitalization (8.24 ± 2.65 days vs. 6.69 ± 2.79 days, *P* = 0.04) (Table 2).

## 4 Discussion

Delayed surgical management of CDC may precipitate severe sequelae, including recurrent cholangitis, obstructive cholestasis, and malignant degeneration. While early diagnosis and timely intervention are paramount for optimizing long-term prognoses, whether radical surgery of CDC should be performed during the neonatal period is still debatable (12, 13, 17). According to current clinical guidelines, in asymptomatic situations, surgical treatment should be postponed until at least three months of age (14). However, neonates with massively dilated cysts are at increased risk of biliary obstruction, hepatic dysfunction, cholangitis, and spontaneous cyst perforation; these complications are often precipitated by the initiation of enteral feeding (15, 18).

Laparoscopic cyst excision and SPCH have become the preferred CDC surgical approach, offering reduced blood loss, fewer postoperative complications, shorter hospital stays, and superior cosmetic outcomes compared with open procedures. This technique has gained widespread acceptance among pediatric surgeons (19–21). Single-port laparoscopic technique is increasingly used in pediatric surgeries, providing additional cosmetic advantages through a single 2–3 cm umbilical incision, further minimizing wound-related complications and postoperative pain (22).

This retrospective comparative study demonstrates that single-port laparoscopic choledochal cyst excision with Roux-en-Y hepaticojejunostomy (SPCH) is technically feasible and safe in neonates. The primary outcomes showed no significant differences between neonatal and older infants and children groups in operative time (230.33 vs. 256.43 min), blood loss (6.43 vs. 6.86 mL), or postoperative complication rates (1/21 vs. 1/49). These findings support the hypothesis that neonatal anatomical characteristics do not compromise the technical execution of SPCH when performed by experienced pediatric surgeons (23).

Several key findings warrant detailed discussion. First, the absence of small-diameter hepaticojejunostomy anastomoses in neonates (0/21 vs. 16/49 in non-neonates, *P* = 0.003) is noteworthy. This finding can be attributed to the predominance of large cyst morphology in neonates (mean diameter 5.49 cm), which facilitates the anastomosis by providing adequate common hepatic duct caliber. Additionally, the less fibrotic tissue and better tissue elasticity in neonates may contribute to easier ductal manipulation. Second, despite comparable operative outcomes, neonates required significantly longer postoperative hospitalization (8.24 ± 2.65 vs. 6.69 ± 2.79 days, *P* = 0.04). This difference likely reflects heightened clinical vigilance in neonatal care—including feeding establishment, jaundice monitoring, and parental education—rather than increased surgical morbidity, as evidenced by similar complication rates between groups. Third, the higher prenatal diagnosis rate in neonates (18/21 vs. 24/49, *P* = 0.004) indicate that neonates in this series represented a selected high-risk population with large, symptomatic cysts, validating the surgical indication criteria used in our center.Our findings are consistent with published literature on neonatal CDC management. Shirota et al. reported that prenatally diagnosed CDC cases often develop symptoms rapidly after birth, supporting early surgical intervention in symptomatic neonates (16). Pan et al. demonstrated the safety of minimally invasive surgery for prenatally diagnosed CDC, with comparable outcomes to older children (13).

The operative time in our neonatal group (230.33 min) is comparable to or shorter than that in reported series of conventional laparoscopic CDC excision in infants, suggesting that the single—port approach does not prolong the operative duration in experienced hands. The absence of bile leakage and the low complication rate (4.8%) in neonates align with the safety profile reported in multiport laparoscopic series. The significantly lower PBM detection rate in neonates (1/21 vs. 22/49, *P* = 0.001) reflects the diagnostic limitations of MRCP in neonates due to immature bile secretion and small-caliber bile ducts, emphasizing the importance of intraoperative cholangiography or careful ductal exploration in this age group.

**Figure 2:**
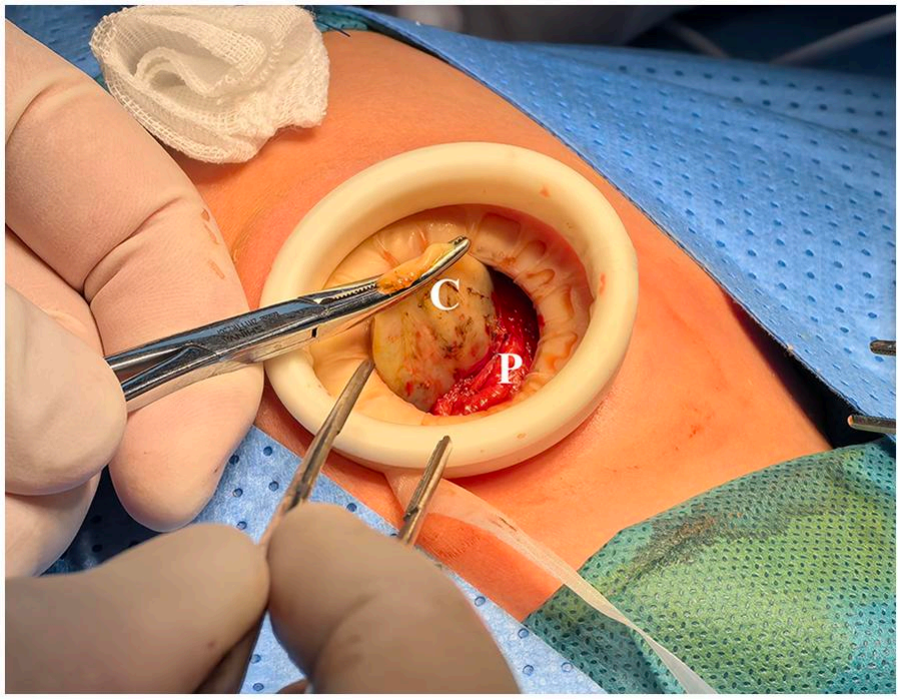
The distal portion of dilated choledochal cysts (C) can be effectively dissected under direct visualization through the umbilical single-port access. This technique, which combines optical magnification with manual dissection, optimizes surgical efficiency by significantly reducing the time required for cyst mobilization, thereby decreasing total operative duration (C, distal choledochal cyst segment; P, superior pancreatic margin).

The technical feasibility of SPCH in neonates can be attributed to specific anatomical advantages. Neonates possess more rounded abdominal cavities and a relatively larger umbilical region relative to body size, which partially mitigates the “chopstick effect” inherent to single-port surgery. For large CDC extending caudally to the umbilical level, the ability to perform distal cyst dissection under direct visualization through the umbilical port (Figure 2) significantly reduces operative time. However, we acknowledge the technical challenges: the narrow working space may restrict instrument triangulation, and limited pneumoperitoneum pressure (8 mmHg) reduces exposure. These factors necessitate advanced laparoscopic skills and thorough understanding of neonatal anatomy. All procedures in this series were performed by experienced pediatric surgeons who had completed the learning curve for both multiport and single-port laparoscopic CDC surgery, highlighting the importance of surgical expertise for safe implementation (23, 24).

Several limitations of this study should be acknowledged. First, the retrospective design introduces inherent selection bias, as neonates undergoing SPCH represented a selected population with large or symptomatic cysts. Second, the modest sample size (21 neonates) may limit statistical power to detect small but clinically meaningful differences in rare complications. Third, the absence of a multiport laparoscopic or open surgery control group precludes direct comparison of SPCH with conventional approaches in neonates. Fourth, the follow-up duration, while sufficient for short-term outcome assessment (median 35 months), may not capture very late complications such as anastomotic stricture developing beyond childhood. Fifth, all procedures were performed by a single surgical team with extensive experience in laparoscopic CDC surgery, potentially limiting generalizability to centers with less experience. Prospective multicenter studies with larger cohorts and longer follow-up are needed to validate these findings and establish standardized protocols for neonatal SPCH.

## 5 Conclusion

This study demonstrates that single-port laparoscopic choledochal cyst excision with Roux-en-Y hepaticojejunostomy is a feasible and safe therapeutic option for neonates with CDC. The procedure offers comparable operative outcomes to older infants and children patients, with the advantages of superior cosmetic results through a single umbilical incision. Neonatal anatomical characteristics, including rounded abdominal cavity and larger umbilical region relative to body size, facilitate technical execution. For neonates with large cysts (≥5 cm) or symptomatic CDC, early SPCH intervention can be performed without increasing procedural risks. Successful implementation requires advanced laparoscopic expertise and meticulous perioperative management. These findings support the consideration of SPCH as a viable minimally invasive approach for carefully selected neonatal CDC patients.

## Data Availability

The original contributions presented in the study are included in the article/Supplementary Material, further inquiries can be directed to the corresponding author/s.
